# Analysis of 16S rRNA gene sequencing data for the taxonomic characterization of the vaginal and the fecal microbial communities in Hanwoo

**DOI:** 10.5713/ab.22.0040

**Published:** 2022-09-02

**Authors:** Soyoung Choi, Jihye Cha, Minji Song, JuHwan Son, Mi-Rim Park, Yeong-jo Lim, Tae-Hun Kim, Kyung-Tai Lee, Woncheoul Park

**Affiliations:** 1 Animal Genomics and Bioinformatics Division, National Institute of Animal Science, RDA, Wanju 55365, Korea

**Keywords:** Feces, Hanwoo, Microbiome, 16S rRNA, Vagina

## Abstract

**Objective:**

The study of Hanwoo (Korean native cattle) has mainly been focused on meat quality and productivity. Recently the field of microbiome research has increased dramatically. However, the information on the microbiome in Hanwoo is still insufficient, especially relationship between vagina and feces. Therefore, the purpose of this study is to examine the microbial community characteristics by analyzing the 16S rRNA sequencing data of Hanwoo vagina and feces, as well as to confirm the difference and correlation between vaginal and fecal microorganisms. As a result, the goal is to investigate if fecal microbiome can be used to predict vaginal microbiome.

**Methods:**

A total of 31 clinically healthy Hanwoo that delivered healthy calves more than once in Cheongju, South Korea were enrolled in this study. During the breeding season, we collected vaginal and fecal samples and sequenced the microbial 16S rRNA genes V3-V4 hypervariable regions from microbial DNA of samples.

**Results:**

The results revealed that the phylum-level microorganisms with the largest relative distribution were *Firmicutes*, *Actinobacteria*, *Bacteroidetes*, and *Proteobacteria* in the vagina, and *Firmicutes*, *Bacteroidetes*, and *Spirochaetes* in the feces, respectively. In the analysis of alpha, beta diversity, and effect size measurements (LefSe), the results showed significant differences between the vaginal and fecal samples. We also identified the function of these differentially abundant microorganisms by functional annotation analyses. But there is no significant correlation between vaginal and fecal microbiome.

**Conclusion:**

There is a significant difference between vaginal and fecal microbiome, but no significant correlation. Therefore, it is difficult to interrelate vaginal microbiome as fecal microbiome in Hanwoo. In a further study, it will be necessary to identify the genetic relationship of the entire microorganism between vagina and feces through the whole metagenome sequencing analysis and meta-transcriptome analysis to figure out their relationship.

## INTRODUCTION

Cattle are important for humankind, and their microbiome research has mainly examined the rumen, small intestine, and other parts of the digestive tract [1]. However, the study of the microbiome in the digestive tract has been limited because it is difficult to collect samples from living animals. Most digestive tract samples have been obtained from slaughtered animals or by using a catheter. Collecting samples of the fecal microbiome is easier than the collection of other samples, and the feces also provide useful biomarkers in real farms and are furthermore known to be related to the host’s health. Accordingly, many studies have used feces samples [2,3].

Beef cattle research has usually been focused on improving meat quality and productivity. Nonetheless, in the beef cattle industrial market, the reproduction rate is important to farmers. Reproductivity is directly influenced by cattle’s reproduction organs. Exogenous factors can contact the reproduction tract because the vagina does not perfectly block these factors. If the host has a disease or encounters other stressful situations, harmful elements can enter, such as germs, and infect the host. A pathogenic infected vagina and uterus decreases the conception rate and can lead to unhealthy calves [4]. In humans, the vaginal microbiome has been examined for primary ovarian failure between normal groups and patient groups, and they showed significantly different microbiome diversity [5]. Recently, animal organ microorganism study was conducted using next generation sequencing (NGS) analysis for confirmation of whole environmental bacterial composition instead of isolation of single strain methods. The microbiome in the reproductive system of cattle has just lately been studied [6], but it is relatively lacking compared to other studies in other organs. Microbiome research of reproductive organs was conducted using vagina and uterus swabs or flushing samples [7,8]. Clemmons [7] reports that the vagina and uterus microbiome in postpartum lactating cows had a high composition of the bacteria phylum *Firmicutes*, and many unknown microorganisms have been observed in the uterus. Furthermore, the relative abundances of microorganisms in the vagina were higher than in the uterus [7]. In dairy cattle, the vaginal microbiome of pre-partum and post-partum heifers were investigated, and particular pathogenic bacteria such as *Escherichia coli* proliferated after parturition [9]. Another study on beef cattle reported that the vagina and uterus bacterial composition changed during the pregnancy period [10]. Research on the microbiome related to disease in the reproductive tract has been mainly reported in dairy cows, and a recent study reported that relative abundances of *Fusobacterium* in metritis groups were higher than in normal groups, and the bacterial composition was also significantly different [6].

In the last few years, human reproduction research has focused on the construction of the baby microbiome influenced by the maternal reproductive organ microbiome. A few studies have found that the maternal gut microbiome and vaginal microbiome can have an impact on newborn health [10], and studies of the relationship between the vaginal and fecal microbiome have also been conducted [11]. Similar research on the cattle microbiome has been carried out. In dairy cows, there was a relationship investigated between the vaginal microbiome of the mother and the fecal microbiome of calves [12]. A study of the beef cattle microbiome revealed that the vaginal microbiome and gut microbiome had co-occurring bacteria, and common vaginal infection bacteria, such as *vaginalis*, were observed in the gut microbiome [13]. Hanwoo cattle were studied using methods of isolated single strains in uterus samples [14]. Methods such as using NGS and a reproductive organ microbiome study in Hanwoo cattle have not been reported to date.

The fecal microbiome has been widely researched. Among them, a lot of research on cattle has recently been reported to discover useful biomarkers. A study of the gastrointestinal (GI) tract microbiome from slaughtered beef cattle reported that the fecal microbiome was related to the GI tract microbiome [15]. Other gut microbiome study investigated changes in the bacterial composition when calves had digestive diseases [16]. In order to improve the health of Hanwoo and generate stable profit for Hanwoo farmers, we need to study healthy Hanwoo cattle’s reproductive microenvironment and discover the relationships with the fecal microbiome to develop a biomarker. However, only a few research studied the characteristics of the vaginal and fecal microbiome in the same cattle simultaneously. There has been no studies in Hanwoo to determine the characteristics of the vaginal microbiome using the 16S rRNA gene sequencing. For this purpose, the present study aims to identify the compositional characteristics and relationship of Hanwoo cattle’s vaginal and fecal microbiome by firstly analyzing the 16S rRNA sequencing, and examining the function of microorganisms. Also, the goal is to examine if fecal microbiome can be used to predict vaginal microbiome in Hanwoo.

## MATERIALS AND METHODS

### Sample collection

Vaginal and fecal samples were obtained from a total of 31 Hanwoo cattle during estrous cycles in Cheongju Province, South Korea. All cattle had a record that one or more calves were born from 2015 to 2018 (Supplementary Table S3). Fecal samples were deposited in a DNA preserved solution swab kit (Noble bio, Hwaseong, Korea) and immediately placed on ice. The vulvar area was wiped clean with a towel and vaginal samples were collected by inserting a long-handled sterile cotton swab (16 cm) into the vagina and rolling in the middle point of the vaginal interior wall surface, to achieve the scrub substances and deposited in a 50 mL conical tube (Becton, Dickinson and Company, Franklin Lakes, NJ, USA) and immediately placed on ice. All samples were stored at −80°C until DNA extraction. Ethics approval was obtained from the National Institute of Animal Science (approval no: NIAS20201979).

### Extract microbial 16S rDNA and next-generation sequencing

DNA was extracted from the feces with a QIAmp PowerFecal DNA kit (QIAGEN, Inc, Germantown, MD, USA) according to the manufacturer’s protocol. DNA extraction from vaginal swabs was performed using a QIAAmp BiOStic Bacteremia DNA Kit (QIAGEN Inc, USA) according to the manufacturer’s protocol. We tore the cotton and put it in served bead tubes (2 mL) for DNA extraction, and then used a MagNa lyser (6,000 rpm, 30 s) for bead-beating three times in the lysis step. Extracted DNA samples were stored at −20°C until the amplification process for sequencing. DNA samples were amplified by the hypervariable regions V3-V4 specific primers (Adaptor/Sequencing primer/Specific locus primer; 341F: 5′-CCTACGGGNGGCWGCAG-3′, 806R: Reverse 5′-ACTACHVGGGTATCTAATCC-3′) of 16S rRNA gene sequence. polymerase chain reaction (PCR) amplification was performed using thermal cycling conditions. Initial denaturation was carried out at 95°C for 3 min, followed by 25 cycles of denaturation at 95°C for 30 s, and a final elongation step at 72°C for 5 min. The PCR products were purified using AMPure XP beads (Beckman Coulter, Nyon, Switzerland). Secondary amplification was conducted using the first PCR amplicon products and attaching an adaptor under the first amplification condition, but only for eight cycles. The DNA quality and product size were assessed on Bioanalyzer 2100 (Agilent, Palo Alto, CA, USA) using DNA 7500 chip. Mixed amplicons sequencing was performed using an Illumina MiSeq with 2×300 bp paired-end reads (Illumina, Inc., San Diego, CA, USA).

### Sequence and statistical analysis

Sequence reads were processed using Quantitative Insight Into Microbial Ecology 2 (QIIME2, 2020.4.) with the default parameter [17]. DADA2 was used for quality filtering and denoising, and low-quality sequences were removed with a quality score (<Q 25) and amplicon sequence variant calling (ASVs) using the qiime dada2-denoise-paired method. ASV feature counts of the 16S rRNA sequence were classified using the naïve Bayes method [18] and the Greengenes (v.13_8) database was used for the assigned taxonomy ID on the alignment with the classification of different levels [19]. Shannon Diversity index, and richness (number of observed features) was calculated using QIIME2 to evaluate alpha diversity. The Kruskal-Wallis test was used to detect statistical differences. Beta diversity was presented by using a principal coordinate analysis (PCoA) and estimated by the phylogenetic distance among samples calculated using unweighted UniFrac dissimilarity based on the phylogenetic tree. The significant differentiation of microbial composition between two groups was assessed by a permutational multivariate analysis of variance (PERMANOVA). The linear discriminant analysis (LDA) effect size (LEfSe) method was conducted to detect any bacterial taxon having a significantly different abundance between vaginal and fecal groups, and visualization was performed using the platform Galaxy (http:huttenhower.sph.harvard.edu/galaxy/) for LEfSe analysis. Statistically significant taxa were reported with LDA scores >4 [20].

The relationships between vaginal and fecal microbiome were estimated by calculating the Spearman correlation (at the genus level). The correlation results were visualized by a ggplot2 in R package version 3.6.3 (R Foundation for Statistical Computing, Vienna, Austria). For all statistical tests, the significance level was set as a p-value<0.05.

### Microbial function prediction using Kyoto encyclopedia of genes and genomes Orthology

Microbial functional profiling was investigated using the relative abundance of assigned bacterial taxa of ASVs. We used Phylogenetic Investigation of Communities by Reconstruction of Unobserved States2 (PICRUSt2) [21] for the predicted microbial function from 16S rRNA data. Using qiime picrust2 full-pipeline, Kyoto encyclopedia of genes and genomes (KEGG) Orthology (KO) was generated from the amplicon sequence variants table and the effect size of KOs was calculated and a comparative analysis between the vagina and feces groups was conducted. KOs with effect size threshold >1 and/or Benjamin-Hochberg adjusted p-value<0.01 (Wilcoxon rank sum test) were identified as distinct KOs using the ALDEx2 [22] package in R. Gene set enrichment analysis (GSEA) [23] was applied to determine the significant enriched metabolic pathway in the KEGG Orthology database for distinct KOs functions using the R package clusterprofiler. Significantly enriched metabolic pathways were shown by a dot plot in R package ggplot2. Statistical analysis and visualization were performed using R package version 3.6.3 (R Foundation for Statistical Computing, Austria).

## RESULTS

### 16S rRNA raw data quality control

A total of 62 samples were collected from Hanwoo cattle in the breeding season. Fecal (n = 31) and vaginal samples (n = 31) were used for microbial DNA extraction and sequencing of the V3 – V4 region of the 16S rRNA gene and generated 5,758,259 pair-end reads using the Illumina NGS sequencing platform. After removing low quality reads (Q score <25) with chimeric reads, 3,592,412 reads (ranging from 9,457 to 124,197 reads) remained using the plugin DADA2 workflow in QIIME2. Vaginal samples that remained ranged from 9,457 to 124,197 reads, with an average of 62,337 reads per sample. Fecal samples that remained ranged from 41,676 to 66,982 reads, with an average of 51,515 reads per sample.

### Bacterial structure and diversity analysis

**Figure 1:**
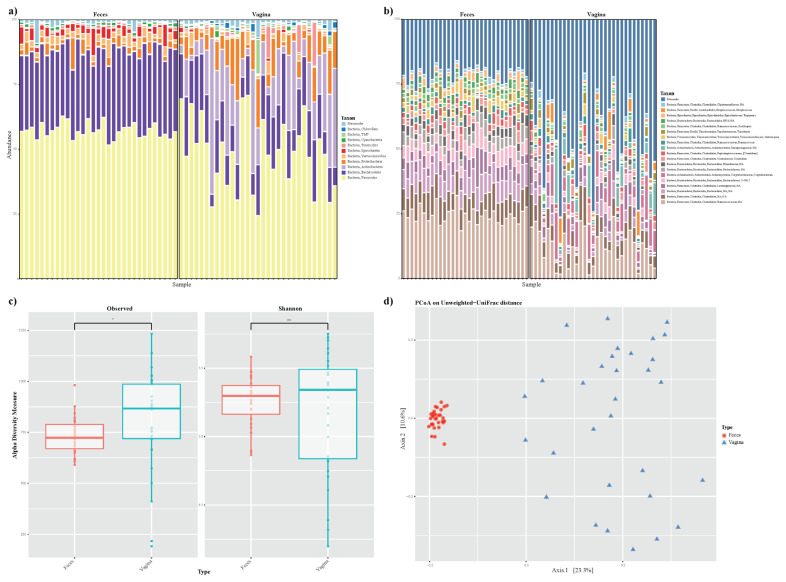
Relative abundance of vaginal and fecal microbiota at the (a) phylum level and (b) genus level. (c) Box plot representation of alpha diversity. Diversity in the vaginal and fecal community was measured using the Shannon index and observed ASVs. (d) Principal coordination analysis (PCoA) using Unweighted Unifrac distance. Beta diversity was used to examine the compositional dissimilarity between vaginal (blue) and fecal microbiota (red). ASV, amplicon sequence variant.

We found a total of 6,595 ASVs in vaginal and fecal samples and conducted taxonomic classification and annotation using the bioinformatics tools QIIME2 with a reference database. In vaginal samples, *Firmicutes* (48%), *Actinobacteria* (18%), *Bacteroidetes* (17%), *Proteobacteria* (9%), *Tenericutes* (2%), and *Verrucomicorbia* (2%) were major abundant bacterial phyla. In fecal samples, *Firmicutes* (57%), *Bacteroidetes* (30%), *Spirochaetes* (3%), *Verrucomicrobia* (3%), *Proteobacteria* (3%), and *Cyanobacteria* (2%) represented more than 90% of all the annotated microbial phyla (Figure 1a). At the genus level, *f_Ruminococcaeae* uncultured (10%), *Corynebacterium* (6%), *o_Clostridiales* uncultured (5%), *f_Intrasporangiaceae* uncultured (3%), *Streptococcus* (2.5%), and *Clostridium* (2.2%) were detected in vaginal samples. In fecal samples, at the genus level, *f_Ruminococcaeae* uncultured (21%), *o_Bacteroidales* uncultured (7.9%), and *o_Clostridiales* uncultured (6.9%) were present (Figure 1b). To identify the microbial diversity of the samples, species evenness (Shannon index) and species richness (observed ASVs) of both samples were evaluated. We did not detect any significant differences in Shannon indices (Figure 1c; Kruskal-Wallis test, p = 0.64). Regarding microbial community richness (e.g., the number of observed ASVs), the vaginal and fecal groups showed significant differences (Figure 1c; Kruskal-Wallis test, p<0.05). We also examined dissimilarity in the bacterial community composition and structure between vaginal and fecal samples. The community structure was analyzed using PCoA plot based on unweighted UniFrac distance matrices. The results showed significant differences in the microbial communities between the vaginal and fecal groups (Figure 1d; PERMANOVA, p>0.001). Furthermore, vaginal samples showed dispersed, whereas those in the fecal samples were clustered.

### Linear discriminant analysis of the vaginal and fecal microbiota

**Figure 2:**
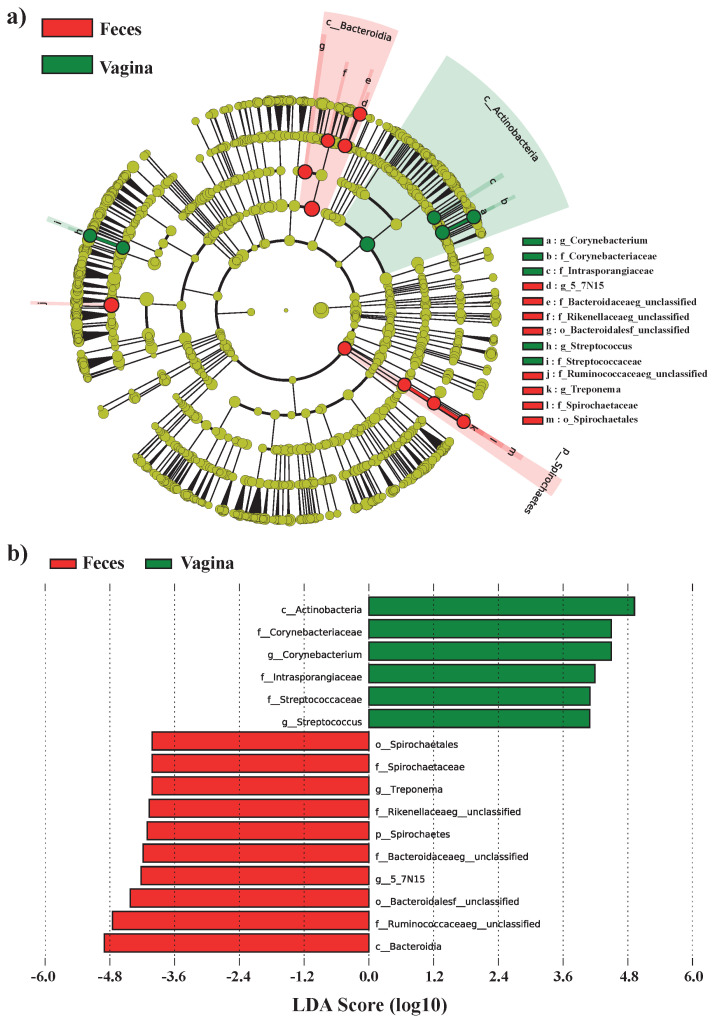
The differentially enriched ASVs in vaginal and fecal groups’ microbiome using the linear discriminant analysis effect size (a, LEfSe). Statistically significant groups were reported with linear discriminant analysis (b, LDA) scores > 4. ASV, amplicon sequence variant.

To identify the significant differences between the vaginal and fecal samples, we performed LDA analysis with effect size measurements (LEfSe) analysis (Figure 2a and 2b; LDA> 4, p<0.05). For the analysis, we used the relative abundance of annotated genera in all samples. A total of six bacterial taxa were significantly abundant in the vaginal group (*c_Actinobacteria*, *f_Corynebacteriaceaea*, *g_Corynebacterium*, *f_Instrasproahgiaceae*, *f_Streptococcaeceae*, *g_Streptococcus*), and 10 bacterial taxa were significantly abundant in the fecal group (*o_Spriochaetales*, *f_Spirochaetaceae*, *g_Treponema*, *f_Rikenellaceae*, p_Spriochaetes, f*_Bacteroidaceae*, *g_5_7N15, o_Bacteroiales*, *f_Ruminococcaeae*, *c_Bacteroidia*).

### Microbial functional metabolic pathway prediction and distinction

**Figure 3:**
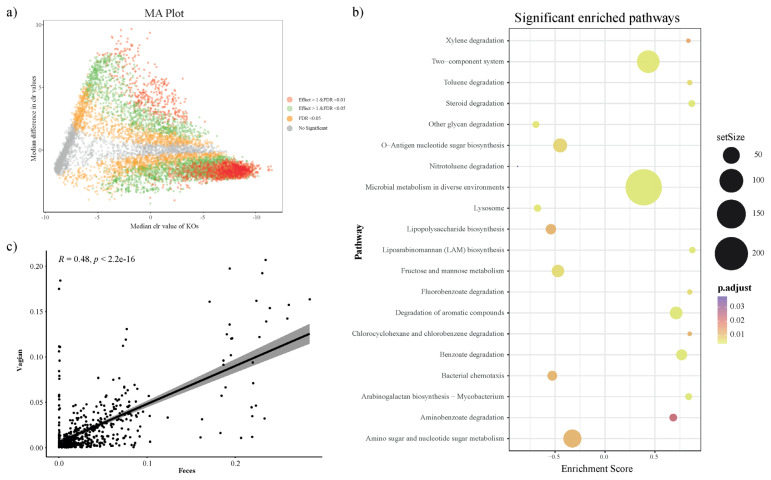
PICRUSt2 KEGG Orthology (KO) prediction, distinction, and gene set enrichment results (a) MA plot showing significant distinct KOs determined by ALDEx2 algorithm between fecal group (n = 31) and vaginal group (n = 31). KOs with BH adjusted p-value<0.01 and effect size >1 (distinct in vaginal group) or effect size <–1 (distinct in fecal) were considered to be significantly distinct. Only KOs with effect size >1 and BH adjusted p-value<0.01 were applied for the subsequent gene set enrichment analysis. (b) Significantly enriched KEGG pathway dot plot. KEGG pathways with adjusted p-value<0.05 in gene set enrichment analysis (GSEA) were considered significantly enriched (Enrichment scores >0: enriched in vaginal group; <0: enriched in fecal group). (c) The relationship between observed genera in vaginal and fecal samples (Spearman, annotated genus). KEGG, Kyoto encyclopedia of genes and genomes.

To investigate the predicted functional ability according to the microbial composition in vaginal and fecal samples, we used the PICRUSt2 plugin in QIIME2, and conducted KO prediction. We used the R package ALDEx2 to detect distinct KOs and determine differences between the vaginal and fecal groups. KOs with a BH adjusted p-value (BH-FDR)<0.01 and/or effect size threshold >1 were considered distinct KOs in both groups. In addition, we applied a MA plot to show the significantly distinct KOs (Figure 3a). The significantly distinct KOs were determined by GSEA using R package clusterprofiler, and significantly enriched KEGG pathways from GSEA presented 20 biological metabolic pathways (Figure 3b; Supplementary Table S1). In the vaginal group, the mainly presented metabolic pathways were ‘Microbial metabolism in diverse environment’, ‘Two component system’, ‘Degradation of aromatic compounds’ and ‘Benzoate degradation’. The fecal group presented biological pathways such as ‘Amino sugar and nucleotide sugar metabolism’, ‘Fructose and mannose metabolism’, and so on.

### Correlation between vaginal and fecal microbiota

We studied the significant differences between vaginal and fecal microbiota. Furthermore, we attempted to find the relationship between vaginal and fecal microbiota composition. Correlation analysis was performed by using the relative abundance of only annotated bacterial genera that were simultaneously present in both groups. First, we performed the correlation analysis using all sample’s vaginal and fecal microbiota. As a result, the vaginal and fecal microbiota were correlated, but they were located as outliers because most genera were in very low abundance (Figure 3c). So next, we checked each sample between vaginal and fecal microbiota using the same method (Supplementary Figure S1 and Supplementary Table S2). However, we identified that vaginal and fecal microbiota in Hanwoo were not significantly correlated.

## DISCUSSION

Recently, in humans, the vaginal microbiome is known to be affected by fecal microbiome [11,13]. Also, the vaginal and uterine microbiome have been examined to determine the characteristics of microbiome and the relationship between microbiota in both organs [24]. However, there has been little study on the bacterial composition of the reproductive tract and its functions in relation to the fecal microbiome in the breeding season of Hanwoo cattle. In this study, we firstly identified the relationship between the vaginal and fecal microbiome of Hanwoo.

In the vagina, *Firmicutes*, *Actinobacteria*, *Bacteroidetes*, and *Proteobacteria* are the large abundant phyla, and in the feces, *Firmicutes*, *Bacteroidetes*, and *Spirochaetes* were the most abundant taxa at the phylum level. These results are consistent with the previous reports of other cattle breeds [6]. However, in humans, the vaginal microbiota has a high relative abundance of *Firmicutes*, *Bacteroidetes*, *Actinobacteria*, and *Fusobacteria* at the phylum level [25]. In the present study, we found the *Ruminococcaeae* uncultured genus, which commonly had the largest relative abundance in the vagina and feces of Hanwoo. In feces, *Ruminococcaeae* had about twofold higher relative abundance than in the vagina. Another study presented that *Ruminococcaeae* has been found usually in the hindgut and feces in beef cattle [1]. Also, it is abundant in the digestive tract of cattle [26]. In the human vagina, *Lactobacillus* is known as the dominant genus [27] but not in the Hanwoo vagina in the present study. Other studies showed that the vaginal microbiome of mammals differs depending on the species [28]. These findings imply that there are interspecies differences in the predominant microorganism that colonized the vagina.

In Beta diversity analysis, the microbial compositions of the vaginal and fecal samples were distinctly different. So, we investigated which microorganisms made difference between the vaginal and fecal samples. In the vagina, we found *Corynebacterium*, *Streptococcus*, and *Intrasporangiaceae* are significantly higher than in the fecal samples. *Corynebacterium*, belonging to the *Actinobacteria* class, other studies confirmed that this microorganism is commonly detected in the reproductive organ of cattle [29]. And *Streptococcus* and *Intrasporangiaceae* were known to present in the other cattle’s vagina, and they were found more frequently in the follicular phase than the luteal phase [8]. *Streptococcus* was identified as a common major microorganism in the vagina of calving cows and the feces of calves [30]. In the feces, we confirmed that *Ruminococcaeae* and *Treponema* were significantly higher than in the vagina. *Ruminococcaeae*, belonging to *Bacteroides*, is a dominant microorganism in the rumen, small intestine, and feces of beef cattle [1] and is also prevalent in the digestive tract of dairy cows [31]. Species of the *Ruminococcaeae* break down cellulose into short-chain fatty acids, which provide nutrients to ruminants [32]. *Treponema*, belonging to the class *Spirochaetales*, was also present in the digestive tract of healthy beef and dairy cattle [1,31].

We predicted bacterial genetic information by PICRUTSt 2.0 based on the microbial community results in 16S rRNA analysis. In the vagina, the most frequently detected metabolic pathway is ‘microbial metabolism in the diverse environment’, which includes related various biological metabolism, i.e., carbohydrate metabolism, energy metabolism, and xenobiotic degradation [33]. Following that, the ‘two-component system’ pathway of bacteria allows them to detect and respond to changes in the environment and cell state, and adapt to such changes [34]. This pathway consists of sensor protein-histidine kinase and response regulator (rarely in eukaryotes). A study of the pathogenic strain ‘*Streptococcus agalactiae*’ identified in the human vagina suggested that this pathway could be used to induce antibiotic, antimicrobial resistance, and mucosal surface colonization in the vagina [35]. In addition, the ‘degradation of aromatic compounds’ pathway was identified in the vagina. Aromatic compounds are one of the most common environmental pollutants, and they are known to be degraded by microorganisms [36]. And the ‘Benzoate degradation’ is interpreted as xenobiotic biodegradation, which refers to exogenous chemicals that are not found in organisms. In a human study, the cervical microbiome of cervical cancer patients was examined using the whole metagenomic shotgun sequencing analysis. The results showed that this metabolic pathway in patients was significantly lower than in the normal group [37]. In addition, the ‘defense mechanism’ pathway among the KO of microbial genes in the uterus is present in the normal group, but not in cervical cancer patients [37]. These metabolic pathways were more prevalent in the vagina than the feces, which indicates that maybe the vagina is more exposed to the outside environment, requiring a higher number of these microorganisms to protect against harmful elements.

In the feces, metabolic pathways related to digestion of nutrients were mainly identified (‘Amino sugar and nucleotide sugar metabolism’, ‘Fructose and mannose metabolism’). Although feces cannot precisely represent the organs responsible for digestion and absorption, it was found that there were functions of microorganisms related to nutrient absorption and metabolism compared to the vagina. In another case, it has been confirmed that the metabolic pathways related to digestion and absorption, increased in the microbiome of calves after weaning, and during growth [38]. These metabolic pathways indicate that microorganisms in the rumen and intestinal tract may be transmitted to feces.

To confirm that the fecal microbiome can explain the vaginal microbiome, we studied the correlation by checking the microorganisms present in both samples. We found a correlation of microbiome between the vagina and feces in all samples. However, when checked by individuals, it was shown that some cows had a relationship, and some cows were not. In humans, it is known that the vaginal microbiome is derived from or influenced by the fecal microbiome [11]. In another study that analyzed the microbial composition of the vagina and feces in beef cattle, it was presumed that the microorganisms could be shared by calculating the angle between the anus and the vagina of cattle based on the results of the same strain [13]. However, in the present study, the analysis was conducted using microorganisms present in both samples, but the correlation was not significant. The results of the microbial correlation between vagina and feces analysis were inconsistent, and it was difficult to explain why only a few cows showed a relationship. Therefore, it is suggested that vaginal and fecal samples are to be analyzed respectively.

In this study, we discovered a significant difference in the bacterial composition between the vaginal and fecal groups, as well as a noticeable difference in the microbial gene prediction results. In addition, through correlation analysis, there was no significant correlation between the two groups. These results provide new information about the understanding of the vaginal and fecal microbiome on Hanwoo and suggest that vaginal and fecal microbiome analysis is to be carried out. In a further study, it will be additionally necessary to identify the genetic correlation of the microorganism between vagina and feces through the whole metagenome sequencing analysis and meta-transcriptome analysis to figure out their relationship.

## SUPPLEMENTARY MATERIAL

**Supplementary Table S1**. Significant enriched KEGG pathways in gene set enrichment results **Supplementary Table S2**. Significant correlation between feces and vagina **Supplementary Table S3**. Information of Hanwoo cattle participating in this experiment **Supplementary Figure S1**. The relationship between observed genera in vaginal and fecal samples (Spearman).
