# Supplementary material for: Analysis of 16S rRNA gene sequencing data for the taxonomic characterization of the vaginal and the fecal microbial communities in Hanwoo
Source: Anim Biosci. 2022 Sep 2;35(11):1808–16. doi: 10.5713/ab.22.0040 (PMC9659453; doi:10.5713/ab.22.0040)
Supplement: Supplementary file 1 [file ab-22-0040-suppl.pdf]

Supplementary table S1. Significant enriched KEGG pathways in gene set enrichment results

ID	Description	setSize	Enrichment Score	BH adjusted P-value
ko01120	Microbial metabolism in diverse environments	240	0.387788426	0.004030602
ko01220	Degradation of aromatic compounds	28	0.712935165	0.004030602
ko00362	Benzoate degradation	21	0.767360215	0.004030602
ko00572	Arabinogalactan biosynthesis - Mycobacterium	9	0.838014726	0.004030602
ko00984	Steroid degradation	9	0.869166029	0.004030602
ko00571	Lipoarabinomannan (LAM) biosynthesis	8	0.874970372	0.004030602
ko02020	Two-component system	91	0.432932417	0.004670673
ko00511	Other glycan degradation	9	-0.692224328	0.0054874
ko04142	Lysosome	10	-0.67611026	0.0054874
ko00364	Fluorobenzoate degradation	6	0.848854962	0.006839171
ko00623	Toluene degradation	6	0.848854962	0.006839171
ko00051	Fructose and mannose metabolism	27	-0.47175007	0.006839171
ko00541	O-Antigen nucleotide sugar biosynthesis	34	-0.449311474	0.007427382
ko00520	Amino sugar and nucleotide sugar metabolism	57	-0.327289834	0.011597272
ko02030	Bacterial chemotaxis	17	-0.528017945	0.011597272
ko00361	Chlorocyclohexane and chlorobenzene degradation	5	0.848207475	0.011597272
ko00540	Lipopolysaccharide biosynthesis	19	-0.541747905	0.011597272
ko00622	Xylene degradation	5	0.835292647	0.013354883
ko00627	Aminobenzoate degradation	11	0.683789063	0.02386127
ko00633	Nitrotoluene degradation	3	-0.875856816	0.037567979

Supplementary table S2. Significant correlation between feces and vagina

**Sample ID**	***R***	**P Value**
No1	0.9	2.20E-16
No2	0.9	2.20E-16
No3	0.85	2.20E-16
No4	0.87	2.20E-12
No5	0.75	1.70E-13
No6	0.95	2.20E-16
No7	0.57	0.00051
No8	0.15	0.4
No9	0.85	2.20E-16
No10	0.73	4.40E-12
No11	0.56	2.30E-05
No12	0.73	1.3E-09
No13	0.38	0.02
No14	0.84	2.20E-16
No15	0.61	4.7E-06
No16	0.71	1.8E-08
No17	0.88	2.20E-16
No18	0.96	2.20E-16
No19	0.9	2.20E-16
No20	0.94	2.20E-16
No21	0.97	2.20E-16
No22	0.56	0.00073
No23	0.77	1.8E-07
No24	0.93	2.20E-16
No25	0.92	2.20E-16
No26	0.85	2.20E-16
No27	0.67	4.1E-06
No28	0.82	2.20E-16
No29	0.74	1.1E-07
No30	0.86	2.40E-17
No31	0.69	3.6E-08

Supplementary table S3. Information of Hanwoo cattle participating in this experiment

Hanwoo ID	Birth	Parity
No1	2015-03-22	4
No2	2016-03-04	3
No3	2016-02-21	3
No4	2016-03-23	3
No5	2016-04-18	2
No6	2016-06-20	3
No7	2016-05-30	3
No8	2016-06-30	3
No9	2017-02-17	2
No10	2017-04-25	2
No11	2017-07-22	2
No12	2018-01-10	1
No13	2017-08-26	2
No14	2017-11-20	1
No15	2017-10-30	2
No16	2018-02-24	1
No17	2018-04-03	1
No18	2018-03-14	1
No19	2018-03-14	1
No20	2018-03-16	1
No21	2018-04-07	1
No22	2018-05-03	1
No23	2018-05-07	1
No24	2018-05-31	1
No25	2018-04-21	1
No26	2018-04-24	1
No27	2018-04-11	1
No28	2018-04-30	1
No29	2018-08-11	1
No30	2018-07-26	1
No31	2018-08-17	1

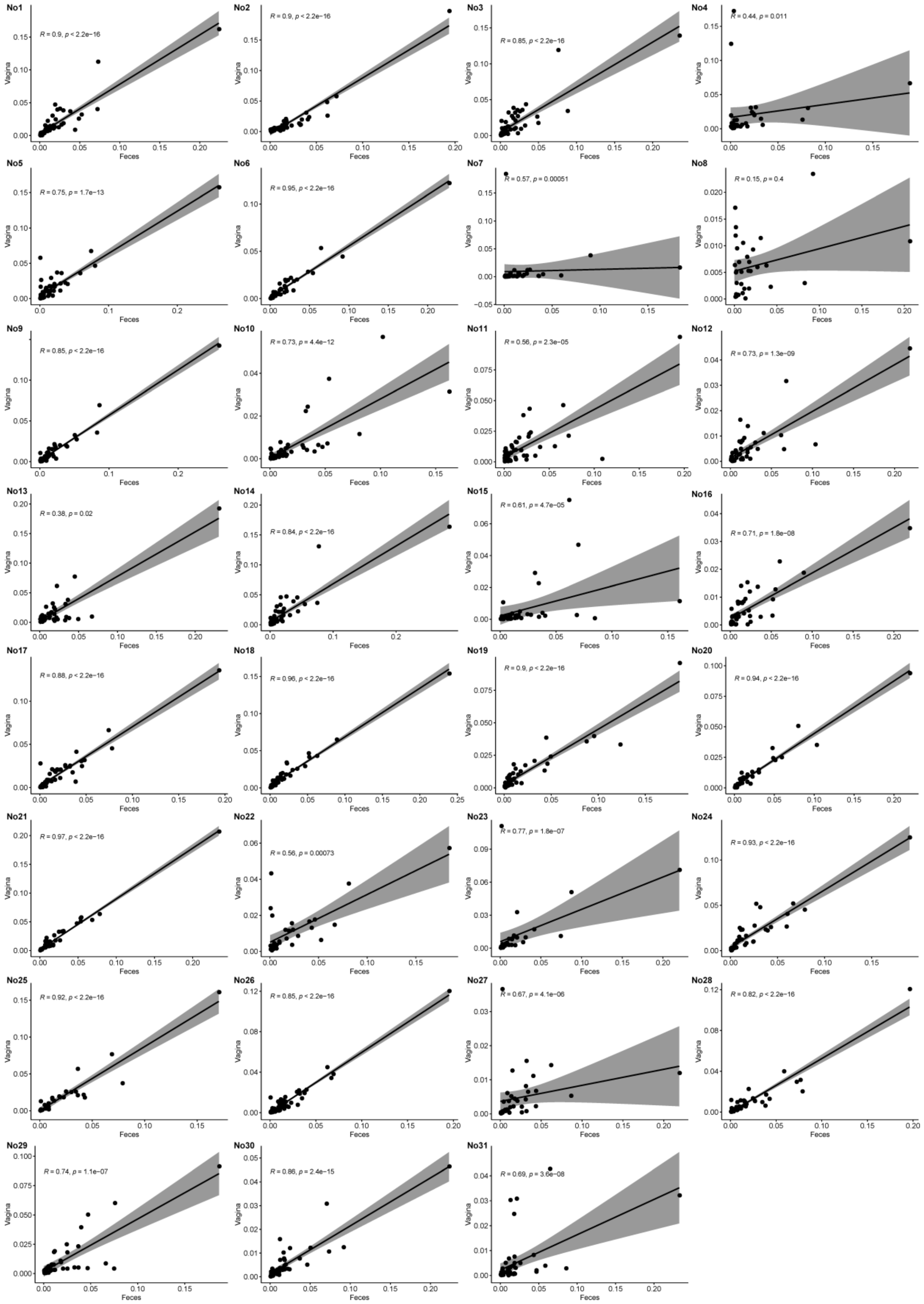

Supplementary figure S1. The relationship between observed genera in vaginal and fecal samples (Spearman).
